# Ethnoveterinary treatment of livestock in Ghana: Cultural importance and consensus on plants used

**DOI:** 10.1016/j.heliyon.2024.e33809

**Published:** 2024-06-28

**Authors:** Maxwell Kwame Boakye, Selase Kofi Adanu, Evans Kwami Buami, Alfred Ofori Agyemang

**Affiliations:** a Department of Environmental Science, Ho Technical University, Ho, Ghana; b Department of Agricultural Engineering, Ho Technical University, Ho, Ghana; c Institute of Traditional and Alternative Medicine, University of Health and Allied Sciences, Ho, Ghana

**Keywords:** *Mangifera indica*, *Elaeis guineensis*, Cultural significance, Perceived efficacy, Localized knowledge

## Abstract

The traditional free-range system of livestock rearing contributes to the socioeconomic well-being of most rural households in Ghana. The traditional management system exposes livestock to a high disease incidence, but healthcare support for animal production is limited. Ethnoveterinary practice is an integral part of livestock treatment, particularly in rural communities where veterinary services are poor. However, documentation of the plants used for ethnoveterinary treatment of livestock is scant in the country. Given the dearth of information on ethnoveterinary treatment, this study aimed to document the cultural significance and level of agreement of plant species used for treating livestock ailments in the Adaklu district. Ethnoveterinary data were collected from 120 respondents using semi-structured questionnaires. Quantitative ethnobotanical indices were used to ascertain the most culturally significant plant species for livestock healing. It was found that 38 plant species were used to treat various livestock ailments. Ethnobotanical indices revealed that *Mangifera indica*, *Elaeis guineensis*, *Khaya senegalensis*, *Spondias mombin*, and *Physalis peruviana* were the most culturally significant plant species for treating livestock ailments. *Mangifera indica* was found to be the most versatile species for treating livestock ailments and had the highest cultural importance (CI). This study reveals the high cultural importance of plants in the Adaklu district to improve livestock healthcare. The perceived efficacy influences the selection and utilization of a resource for folk medicine. The study recommends isolating and characterizing the active compounds in the most culturally significant plants and testing the properties on the medical conditions attributed to these plants.

## 1 Introduction

Livestock production is a significant economic activity in Ghana and provides a vital source of income, assets, and food and nutritional security for households [1,2]. The predominant method for raising livestock in the nation is still the traditional extensive production system, or free-range system, which permits animals to graze without limitations [[2], [3], [4]]. The management system exposes scavenging animals to dangerous conditions, such as bacterial, viral, and parasitic pathogens that increase their susceptibility to infections [5,6]. Accordingly, a high disease incidence is associated with Ghana's free-range system of producing livestock, and it has become a significant obstacle that undercuts benefits [4,[6], [7], [8]]. In order to address the disease-related morbidity in livestock, proper healthcare provision is therefore required. Traditional medicine methods, often known as ethnoveterinary care, are frequently used to enhance animal healthcare.

Ethnoveterinary treatment of livestock diseases is widely practiced in other African countries [[9], [10], [11], [12], [13], [14], [15], [16], [17]] and other countries worldwide [[18], [19], [20], [21]]. In remote and undeveloped areas, there is a lack or limited access to modern veterinary services to cater to livestock healthcare needs, and ethnoveterinary remedies remain a prominent complementary medical practice for treating diseases [10,18]. The use of ethnoveterinary medicine is an integral part of veterinary services for the treatment of livestock in Ghana, and various natural-based products have been documented to be used to treat livestock ailments [[22], [23], [24], [25], [26], [27]]. The perceived effectiveness in treating particular ailments, availability and accessibility of plant resources, and cultural practice are the main factors fueling the reliance on ethnoveterinary practice in Ghana [25].

Plant-based materials for treating livestock are an essential component of Ghana's free-range production system. Despite the acknowledged use of plant-based materials for livestock treatment in the country, there is a dearth of comprehensive information regarding the plants species used for treating livestock ailments. By documenting plant species, ethnoveterinary medicine plays a vital role in maintaining traditional knowledge about therapeutic plants and preserving them for future generations [10]. In addition to offering a chance for new drug discovery from beneficial natural products [21], ethnoveterinary inventory can highlight the well-known species of interest to communities to assess the threat to conservation accurately [[28], [29], [30]].

The Guinea Savannah zone - the largest known area for livestock production [31] is where most Ghanaian ethnoveterinary studies were conducted. The Volta region is becoming increasingly significant for livestock production, particularly in the savanna areas, but little is known about the ethnoveterinary practices associated with free-range livestock production in the region. Access to veterinary services for animal production limits the region's free-range livestock production systems, and, like other parts of the nation, ethnoveterinary practice is the standard approach to animal health care. This study sought to identify the plant species that free-range producers use to treat livestock ailments and to document the cultural significance of these species as well as the degree of agreement regarding their application. The study hypothesizes that the rural nature of the study area, coupled with limited access to veterinary services and a high prevalence of free-range animal husbandry systems, makes livestock caretakers knowledgeable about plant species for ethnoveterinary practice.

## 2 Materials and methods

### 2.1 Study area

The study was conducted in Ghana's Volta Region's Adaklu District, which is situated at latitudes 00°20′1°E and 0.33361°E and longitudes 06°41′1°N and 6.68361°N. According to the Ghana Statistical Service [32], the study area is bordered to the east by Ho-West, to the south by North-Tongu District, to the north by Agotime-Ziope District, and to the east by Akatsi-North District. There are 38,649 people living in the Adaklu District, which has a total land area of 810 km^2^ [33]. The district is entirely rural and most of the population raise livestock and cultivate crops for subsistence. A fifth of the district's total land area is devoted to livestock production, which is essential to the livelihood of the local populace. The use of veterinary services is influenced by small herd sizes, the cost of services, and the lack of access to veterinary officers in rural areas [3]. The district is ideally suited to investigate ethnoveterinary practices because of its rural nature, small herd sizes from subsistence farming practice, and high prevalence of free-range animal husbandry systems. Local communities have been found to rely on traditional medicine to treat human ailments [34] and ethnoveterinary treatment for free-range chicken diseases [35]. It is expected that a significant amount of livestock healthcare will be provided by ethnoveterinary practices in the district.

### 2.2 Sampling procedure and data collection

There are 12 operational areas in the study district that provide agricultural extension services. The extension service staff of Agricultural Services Department helped identify informants in the 12 zones. The criteria used to select the informants included herd size (no less than 10), years of experience producing livestock on a free-range basis (minimum of 10 years), recognition of complementary ethnoveterinary practices (since none of them solely rely on natural materials), and responsibility for animal health and disease management (necessarily animal property rights). Ten informants were chosen from each of the 12 zones based on these specifications. The study's purpose was explained to the community leaders who gave their informed consent to undertake the research in their community.

Semi-structured interviews were used in March and April of 2023 to gather ethnoveterinary information from the study informants. The informants were primarily questioned about local plant names, parts used, preparation and application techniques, and ailments treated. Sheep and goats dominate the household traditional system small ruminants’ production among households in Ghana [1,2], and the questions were limited to these two livestock. The respondents were motivated through verbal prompts and probes to elicit information. Most respondents who could not speak English used the Ewe language, spoken most frequently in the district, as their lingua franca during the interviews. The research assistants were all proficient in both English and Ewe.

To identify plant species, the local names that the respondents had mentioned were compared to those found in the literature [36,37]. Additionally, by comparing the collected plant materials with voucher specimens at the University of Health and Allied Sciences Institute of Traditional and Alternative Medicine (ITAM), their identity was confirmed. The names and authority of plant species were established using electronic databases Plants of the World Online (https://powo.science.kew.org).

### 2.3 Data analysis

The following ethnobotanical indices were identified for each plant species: Use Report (UR), Cultural Importance (CI), Frequency of Citation (FC), Number of Uses (NU), Relative Frequency of Citation (RFC), and Relative Importance Index (RI) using the ethnobotany R package version 4.3.1. According to Ref. [38], the UR values for each species account for the total number of uses within each use category as well as the number of informants who mention each use category for the species. According to Ref. [39], the cultural importance (CI) index determines the cultural importance index for every species in the data set. Prance et al. [38] defined the number of uses (NU) per species as the total of all categories deemed useful for a given species and the frequency of citation (FC) per species as the total of informants citing a use for the same species in the dataset. The relative frequency of citation (RFC) determines the significance of every species based on the number of informants who reported using it. The relative importance (RI) index calculates the relative importance of each species in the data set, considering only the use categories [39].

#### 2.3.1 Informant agreement ratio

The degree of agreement among respondents regarding plant species used to treat a particular livestock ailment was measured using the informant agreement ratio (IAR). Trotter and Logan's [40] original formula was interpreted as follows in equation (1):

(1) $$IAR=\frac{Nur-Na}{Nur-1}$$

where *Nur* = is the total number of use reports recorded for a given plant species, and *Na* = is the total number of illnesses a specific plant species has treated. The IAR ranges from 0 (where there is no agreement or consensus regarding the livestock ailments a plant species treats) to 1 (where there is agreement regarding the livestock ailments a plant species treats).

To gain insight into the livestock ailments that plant species were used to treat, a word cloud visual representation of word frequency was employed using WordItOut. The size of the words corresponds to how frequently the informants brought up the terms.

## 3 Results

**Fig. 1:**
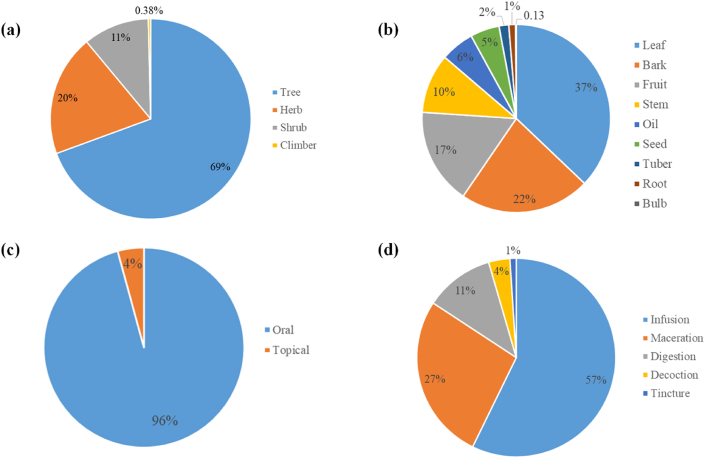
The life form (a), plant parts used (b), mode of administration (c) and preparation of plants used (d) for the treatment of livestock in the Adaklu district.

Male informants who raised sheep and goats in their backyards made up the entire study population. It was found that 38 plant species were used to treat 32 ailments in livestock. The identified species represented 23 botanical families (Table 1). The Asteraceae family was the most represented, with six plant species (26.09 %) followed by the Anacardiaceae, Malvaceae, and Solanaceae, with three species (13.04 %) each representing these families. Two plant species (8.69 %) represented the families of Fabaceae, Lamiaceae, Maliaceae, and Poaceae. One plant species (4.35 %) represented each of the other families (Table 1). Based on the International Union for Conservation of Nature (IUCN) Red List Threatened Species, most of the species were found to be of Least Concern (LC) (n = 20; 52.63 %), Not Evaluated (NE) (n = 13; 34.21 %), Data Deficient (DD) (n = 3; 7.89 %), Vulnerable (VU) and Endangered (EN) (n = 1; 2.63 %, each). Trees ranked highest among the plant life form (n = 542; 69.40 %), followed by herbs (n = 153; 19.59 %), shrubs (n = 83; 10.63 %), and climbers (n = 3; 0.38) (Fig. 1a). The leaf was mentioned the most (n = 290; 37.13 %) out of all the plant parts that were found to be used for treating livestock illnesses. Other plant parts that were mentioned were the bark (n = 175; 22.41 %), fruit (n = 129; 16.52 %), stem (n = 80; 10.24 %), oil (n = 45; 5.76 %), seed (n = 39; 4.99 %), tuber (n = 13; 1.66 %), root (n = 9; 1.15 %), and bulb (n = 1; 0.13) (Fig. 1b). The two most popular administration methods were oral (n = 748; 95.77 %), followed by topical (n = 33; 4.23 %) (Fig. 1c). Infusion (n = 447; 57.23 %), maceration (n = 211; 27.02 %), digestion (n = 88; 11.27), decoction (n = 27; 3.46 %), and tincture (n = 8; 1.02 %) were the most often used methods of preparation (Fig. 1d).

**Table 1:** Plant species used for the treatment of livestock ailments in the Adaklu District with their ethnobotanical indices.

Family	Scientific name	Local name	Voucher ID	Conservation status	URs	CI	FCs	NUs	RFCs	RIs	IAR
Anacardiaceae	*Mangifera indica* L.	Mango	UHAS/ITAM/2021/SB009	DD	123	1.025	98	17	0.817	0.985	0.869
Arecaceae	*Elaeis guineensis* Jacq.	Deti	UHAS/ITAM/2023/L001	LC	101	0.842	101	14	0.842	0.912	0.870
Meliaceae	*Khaya senegalensis* (Desr.) A.Juss.	Logo	UHAS/ITAM/2021/SB007	VU	97	0.808	62	10	0.517	0.601	0.906
Anacardiaceae	*Spondias mombin* L.	Akuko	UHAS/ITAM/2023/FR001	LC	88	0.733	80	10	0.667	0.690	0.897
Solanaceae	*Physalis peruviana* L.	Totototo	UHAS/ITAM/2023/L002	LC	74	0.617	74	6	0.617	0.543	0.932
Caricaceae	*Carica papaya* L.	Adiba	UHAS/ITAM/2023/S001	DD	60	0.500	60	6	0.500	0.474	0.915
Meliaceae	*Azadirachta indica* A.Juss.	Liliti	UHAS/ITAM/2023/L003	LC	42	0.350	42	9	0.350	0.473	0.805
Euphorbiaceae	*Manihot esculenta* Crantz	Agbeli	UHAS/ITAM/2023/L004	DD	42	0.350	42	9	0.350	0.473	0.805
Moringaceae	*Moringa oleifera* Lam.	Babatsi, Yevutsi	UHAS/ITAM/2021/L011	LC	37	0.308	37	10	0.308	0.477	0.750
Malvaceae	*Adansonia digitata* L.	Adido	UHAS/ITAM/2021/SB003	NE	35	0.292	12	3	0.100	0.148	0.941
Malvaceae	*Sida alba* L.	Dameadame	UHAS/ITAM/2023/L005	NE	11	0.092	11	4	0.092	0.172	0.700
Solanaceae	*Solanum aethiopicum* L.	Agbitsa	UHAS/ITAM/2023/FR002	NE	11	0.092	11	2	0.092	0.113	0.900
Poaceae	*Zea mays* L.	Ebli	UHAS/ITAM/2023/L006	LC	7	0.058	7	3	0.058	0.123	0.667
Lamiaceae	*Ocimum basilicum* L.	Dzeviti, Ahame	UHAS/ITAM/2021/AP004	NE	7	0.058	7	7	0.058	0.241	<0.000
Celastraceae	*Maytenus senegalensis* (Lam.) Exell	Wotsi	UHAS/ITAM/2023/L007	LC	6	0.050	6	3	0.050	0.118	0.600
Malvaceae	*Abelmoschus esculentus* (L) Moench	Fetri	UHAS/ITAM/2023/L008	NE	4	0.033	4	2	0.033	0.079	0.667
Musaceae	*Musa × paradisiaca* L.	Abladzo	UHAS/ITAM/2023/L009	NE	4	0.033	4	3	0.033	0.108	0.333
Solanaceae	*Capsicum frutescens* L.	Atadi	UHAS/ITAM/2021/FR006	LC	3	0.025	3	1	0.025	0.044	1.000
Sapindaceae	*Blighia sapida* K.D.Koenig	Atsiatsi	UHAS/ITAM/2023/L010	LC	2	0.017	2	2	0.017	0.069	<0.000
Poaceae	*Bambusa vulgaris* Schrad. ex J.C. Wendl.	Pampro	UHAS/ITAM/2023/FR003	NE	2	0.017	2	2	0.017	0.069	<0.000
Asteraceae	*Ageratum conyzoides* L.	Gbomadu	UHAS/ITAM/2021/R002	LC	2	0.017	2	2	0.017	0.069	<0.000
Asteraceae	*Gymnanthemum amygdalinum (Delile) Sch.Bip*	Gboti	UHAS/ITAM/2023/L011	NE	2	0.017	2	2	0.017	0.069	<0.000
Dioscoreaceae	*Dioscorea esculenta* (Lour.) Burkill	Te	UHAS/ITAM/2023/L012	LC	2	0.017	2	2	0.017	0.069	<0.000
Phyllanthaceae	*Bridelia ferruginea* Benth.	Akamiti	UHAS/ITAM/2023/SB001	LC	2	0.017	2	2	0.017	0.069	<0.000
Asteraceae	*Acanthospermum hispidum* DC.	Deadzoleamengo	UHAS/ITAM/2023/L013	LC	2	0.017	2	2	0.017	0.069	<0.000
Bignoniaceae	*Spathodea campanulata* P. Beauv.	Adatsigo	UHAS/ITAM/2023/L014	LC	2	0.017	2	1	0.017	0.039	1.000
Araceae	*Anchomanes difformis* (Blume) Engl.	Dorli	UHAS/ITAM/2023/L015	LC	2	0.017	2	1	0.017	0.039	1.000
Fabaceae	*Tamarindus indica* L.	Fortsi	UHAS/ITAM/2023/L019	LC	1	0.008	1	1	0.008	0.034	–
Cucurbitaceae	*Momordica charantia* L.	Kakle	UHAS/ITAM/2021/AP003	NE	1	0.008	1	1	0.008	0.034	–
Lamiaceae	*Tectona grandis* L.f.	Teak	UHAS/ITAM/2023/L016	EN	1	0.008	1	1	0.008	0.034	–
Apocynaceae	*Cascabela thevetia* (L.) Lippold	Azibizaba	UHAS/ITAM/2023/L017	LC	1	0.008	1	1	0.008	0.034	–
Anacardiaceae	*Anacardium occidentale* L.	Yevutsa	UHAS/ITAM/2023/SB002	LC	1	0.008	1	1	0.008	0.034	–
Arecaceae	*Cocos nucifera* L.	Agone	UHAS/ITAM/2021/FR008	NE	1	0.008	1	1	0.008	0.034	–
Plantaginaceae	*Scoparia dulcis* L.	Gbeveve/Nugbe	UHAS/ITAM/2023/SB003	NE	1	0.008	1	1	0.008	0.034	–
Boraginaceae	*Heliotropium indicum* L.	Koklo totsu	UHAS/ITAM/2023/L017	NE	1	0.008	1	1	0.008	0.034	–
Fabaceae	*Phaseolus vulgaris* L.	Ayi	UHAS/ITAM/2023/S002	LC	1	0.008	1	1	0.008	0.034	–
Amaryllidaceae	*Allium cepa* L.	Sabala	UHAS/ITAM/2021/BB001	NE	1	0.008	1	1	0.008	0.034	–
Asteraceae	*Taraxacum officinale* F.H. Wigg.	Aŋͻto	UHAS/ITAM/2023/L018	LC	1	0.008	1	1	0.008	0.034	–

UR = use report, CI = cultural importance, FC = frequency of citation, NU = number of uses, RFC = relative frequency of citation, RI = relative importance, IAR = informant agreement ration.

DD = data deficient, NE = not evaluated, LC = least concern, VU = vulnerable, EN = endangered.

Table 1 displays the highest use report (UR) for *M. indica* (n = 123) among the 38 plant species used to treat livestock ailments. *E. guineensis* (n = 101), *K. senegalensis* (n = 97), *S. mombin* (n = 88), *P. peruviana* (n = 74), and *C. papaya* (n = 60) were the next highest URs. For 11 species, there was a single-use report, and for nine species, there were two use reports. In line with the UR, *M. indica* (n = 1.025) was found to have the highest cultural importance (CI) for treating livestock ailments followed by *E. guineensis* (n = 0.842), *K. senegalensis* (n = 0.808), *S. mombin* (n = 0.733), *P. peruviana* (n = 0.617), and *C. papaya* (n = 0.500). FC was highest in *E. guineensis* (n = 101), *then M. indica* (n = 98), *S. mombin* (n = 80), *P. peruviana* (n = 74), *K. senegalensis* (n = 62), and *C. papaya* (n = 60). *M. indica* had the greatest number of uses (NU) for treating various categories of livestock ailments (n = 17), followed by *E. guineensis* (n = 14), *K. senegalensis, S. mombin, and M. oleifera* (n = 10; each), *A. indica, and M. esculenta* (n = 9; each).

*E. guineensis* had the highest RFC value (0.842), and the next highest values were 0.817 for *M. indica,* 0.667 for *S. mombin,* 0.617 for *P. peruviana,* 0.517 for *K. senegalensis,* and 0.500 for *C. papaya. M. indica* (0.985) was found to have the highest (RI), followed by *E. guineensis* (0.912), *S. mombin* (0.690), *K. senegalensis* (0.570), *P. peruviana* (0.543), *M. oleifera* (0.477), *and C. papaya* (0.474) (Table 1).

**Fig. 2:**
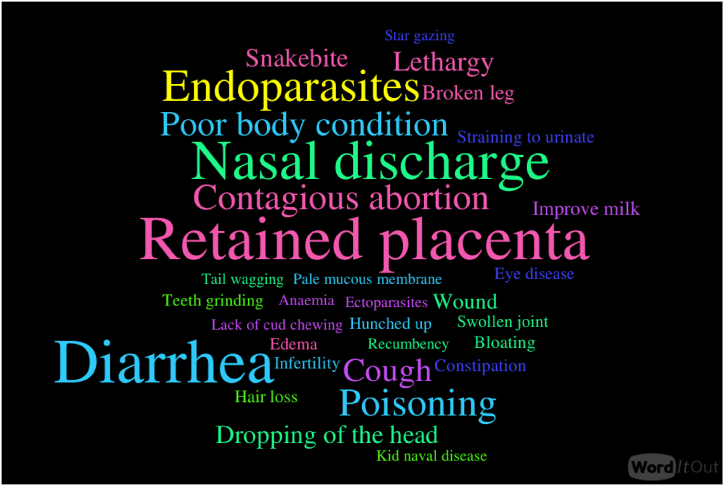
Word cloud of the livestock ailments mentioned by respondents to be treated with plant-based material. Maximum mentions are 114 for retained placenta and a minimum of 1 for tail wagging.

Retained placenta (n = 114) was the most frequently mentioned illness, followed by diarrhea (n = 113), nasal discharge (n = 98), endoparasites (n = 74), and poisoning (n = 58). The condition mentioned the least (n = 1) was tail wagging. A visual presentation of the livestock ailments identified to be treated in this study is presented in Fig. 2. *C. frutescens*, *S. campanulata*, and *A. difformis* (IAR = 1) had the highest IAR (and thus consensus) for the treatment of livestock ailments and were used explicitly for broken legs, lethargy, and nasal discharge, respectively. *A. digitata* recorded the second highest consensus (IAR = 0.941) and was followed by *P. peruviana* (IAR = 0.932), *C. papaya* (IAR = 0.915), and *K. senegalensis* (IAR = 0.906). The supplementary sheet provides the contributions of the 38 plant species to the CI and IAR for treating 32 specific ailments.

## 4 Discussion

The number of plant species recorded in this study for the treatment of livestock ailments was greater than the previous studies on the ethnoveterinary treatment of livestock in Ghana [23,26,41]. The sociocultural knowledge of people living in a particular locality informs the selection and application of medicinal plants for the ethnoveterinary treatment of livestock diseases [9,11,17,42]. Differences in ecological zones also result in variations in plant species and their application for ethnoveterinary purposes [11]. Previous ethnoveterinary studies mentioned were undertaken in the Northern part of the country, which has a different sociocultural group and belongs to a different ecological zone. The variation in the number of plant species found in this study compared to other studies in Ghana may be explained by sociocultural differences and variations in ecological zones.

The number of plant species used for ethnoveterinary purposes in this study compares favorably with similar ethnoveterinary inventories in sub-Saharan African countries. The number of plant species identified to be used for treating livestock ailments was 38 in Mozambique [14], 31 and 30 by Refs. [43,44], respectively, in Nigeria, 26 in Burkina Faso [17], 15 in Namibia [16], 12 in South Africa [15]. However, Tchetan et al. [11] study identified 101 plant species to be used in the ethnoveterinary treatment of livestock diseases in the Benin Republic. The differences in plant species diversity between the two studies can be attributed to the coverage area. While this study covered only one administrative district and ecological zone, Tchetan et al. [11] study was national in focus and covered all the ecological zones in the country. Indeed [11], study recorded a higher plant species diversity relative to similar studies in Benin with limited coverage area.

The dominance of the Asteraceae family was consistent with these studies [19,45]. Trees as the most dominant life forms for treating livestock diseases was consistent in other studies [15,17] and justified by their availability throughout the year, irrespective of the season. The leaf as the most used plant part was consistent with the [[14], [15], [16], [17],^20^,^43^,^44^] study findings. The ease of collection and modes of different applications are attributes that facilitates leaf's widespread use in ethnoveterinary medicine [17]. This study's high preference for oral administration was consistent with [16,45]. The oral and topical routes allow rapid physiological reactions, increasing ethnoveterinary medicine's curative power [10].

The suite of livestock diseases identified in this study was consistent with other studies in Ghana [23,26,41] and other studies on livestock disease treatment with ethnoveterinary care [11,16,19,43,44]. The plant species used to treat specific livestock ailments were consistent with previous research in Ghana and other countries. The range of illnesses that *M. indica* treats was consistent with their ethnoveterinary application in prior researches Nigeria and Benin Republic [12,46]. *K. senegalensis* ethnoveterinary application for the treatment of ailments in this study has been mentioned in previous studies in Ghana [26,41] and other countries in West Africa [11,13,44,46,47]. *G*. *amygdalinum* for the treatment of retained placenta has been recorded in a previous study in Ghana by Ansah and Nagbila [41]. The application of *A. indica* to treat wounds was consistent with Ayeni and Basiri's [44] study in Nigeria. The use of C. papaya and *K. senegalensis* in this study's endoparasite treatment is supported by Ref. [43]. The use of *E*. *guineensis* oil for treating ingested poison has been recorded in folk traditional medicine [48,49] and for livestock in Nigeria [44], and free-range chicken in Ghana [35]. The application of *S. mombin* in the treatment of reproductive diseases was in line with the results of Tchetan et al. [11] in Benin Republic. *P. peruviana*, like *S. mombin*, was valued for its ability to heal reproductive disorders. This study showed a high CI and IAR for *P. peruviana*, contrary to Ref. [50] assertion that it is rarely used for ethnoveterinary purposes.

*M. indica*, *E guineensis*, *K. senegalensis*, *S. mombin*, *P. peruviana*, and *C. papaya* high CI values indicate that these plant species are frequently used in treating livestock ailment and hold a high level of cultural importance as a source of plant-based material. It has been suggested that a CI value of more than one indicates that the local communities use the particular resource to treat various medical conditions [34,35,51,52]. The high CI for *M. indica* implies that it is extremely important for treating livestock ailments. *M. indica* was the most medicinally versatile plant species for ethnoveterinary purposes and was highly valued for treating nasal discharge and diarrhea. The informant agreement ratio (IAR) indicates the homogeneity of ethnobotanical information [53]. Plant species with a high IAR suggest that, because of their perceived effectiveness, they have become essential to local cultural knowledge [53]. Despite having the highest IAR, *C. frutescens*, *S. campanulata*, and *A. difformis* are considered culturally fringe knowledge because of their below average mentions, based on Heinrich et al. [54] assertion. The above-average mentions of *A. digitata*, *P. peruviana*, *C. papaya*, *K. senegalensis, S. mombin*, *E. guineensis****,*** *and M. indica* indicate that they are all essential components of local cultural knowledge; their high IAR may reflect their perceived cultural efficacy in treating livestock ailments.

The choice and application of a resource for folk medicine are influenced by the perceived efficacy of that resource within a culture [53]. Thomas et al. [55] assert that the reference does not imply the local community's view of the effectiveness of traditional medicine in treating a specific ailment. Heinrich et al. [54] state that species mentioned infrequently or less frequently may not be useful for treating the condition(s) mentioned, which may be considered a part of the fringe culture or may have become obsolete due to cultural adaptation. Most plant species have citation rates that are below average, which could mean that they are part of cultural folklore and do not work well for treating specific ailments. This could be the case concerning the below average citations of plant species, which lead to low CI and homogeneity in their use. Despite being listed in the traditional pharmacopeia, these plant species might not be useful for treating or easing symptoms.

Compared to earlier studies, most of the plant species in this study were more widespread in their applications. The cultural transmission of ethnobotanical knowledge could account for the variations in the uses of plants in this study as opposed to earlier research. Intergenerational transmission is the primary way of knowledge about how a resource is handed down in small local communities [56,57]. The majority of knowledge comes primarily from family or community members. Family members verbally transmitting their knowledge of using medicinal plants for healing over several generations is the most common way folk medical information is passed down in Ghana [[58], [59], [60]]. Comparing *M. indica* to other studies, its high versatility may be explained by localized knowledge. The low CI of *G*. *amygdalinum*, which is highly valued for ethnoveterinary purposes in Ghana [23] and other countries [40,[61], [62], [63]], may be explained similarly by disparities in knowledge. The variation can be attributed to localized knowledge about their ethnoveterinary significance. The knowledge systems are not standardized, so there is variation in how they are applied, even for the most prevalent species.

## 5 Conclusion

This study focused on the cultural importance and level of agreement on using plant-based materials in treating livestock ailments. A high degree of agreement, relative importance, and cultural significance were found for certain plant species when treating livestock ailments. The high ethnobotanical indices indicate that ethnoveterinary practices contribute to the health improvement of livestock animal production in the Adaklu district. The most culturally significant species for treating livestock ailments were *M. indica*, *E. guineensis*, *K. senegalensis*, *S. mombin*, *P. peruviana*, and *C. papaya.* The culturally significant plants found in this study may contain active compounds with medicinal value, as perceived efficacy influences the selection and use of resources for folk medicine. The study recommends isolation and characterization of the active compounds in the most culturally significant plants and testing the efficacy against medical conditions attributed to these plants. Based on the IUCN Red List of Species, only *K. senegalensis* is considered a conservation concern among the culturally significant plants.

## Funding statement

This research did not receive any specific grant from funding agencies in the public, commercial, or not-for-profit sectors.

## Additional information

No additional information is available for this paper.

## Consent to participate

Informed consent was obtained from all individual participants included in the study.

## Ethics statement

The Ho Technical University Ethics Committee granted ethical approval for the study (HTU/2023-020).

## Data availability statement

The authors confirm that the data supporting the findings of this study are available within the article and its supplementary material.

## CRediT authorship contribution statement

**Maxwell Kwame Boakye:** Writing – review & editing, Writing – original draft, Methodology, Investigation, Formal analysis, Conceptualization. **Selase Kofi Adanu:** Writing – review & editing, Methodology, Investigation, Conceptualization. **Evans Kwami Buami:** Writing – review & editing, Methodology, Investigation, Conceptualization. **Alfred Ofori Agyemang:** Writing – review & editing, Methodology, Formal analysis, Data curation, Conceptualization.

## Declaration of competing interest

The authors declare that they have no known competing financial interests or personal relationships that could have appeared to influence the work reported in this paper.

## References

[1] Abdulai I.A. (2022). Rearing livestock on the edge of secondary cities: examining small ruminant production on the fringes of Wa, Ghana. Heliyon.

[2] Adams F., Ohene-Yankyera K., Aidoo R., Wongnaa C.A. (2021). Economic benefits of livestock management in Ghana. Agric. Econ..

[3] Adams F., Ohene-Yankyera K. (2015). Determinants of small ruminant farmers decision to participate in veterinary services in Northern Ghana. J. Vet. Med. Anim. Health.

[4] Turkson P.K., Naandam J. (2006). Constraints to ruminant production in east Mamprusi district of Ghana. Ghana J. Agric. Sci..

[5] Åkerfeldt M.P., Gunnarsson S., Bernes G., Blanco-Penedo I. (2021). Health and welfare in organic livestock production systems—a systematic mapping of current knowledge. Org. Agr.

[6] Squire S.A. (2019).

[7] Adams F. (2015).

[8] Turkson P.K., Naandam J. (2003). Assessment of veterinary needs of ruminant livestock owners in Ghana. Prev. Vet. Med..

[9] Oda B.K., Lulekal E., Warkineh B., Asfaw Z., Debella A. (2024). Ethnoveterinary medicinal plants and their utilization by indigenous and local communities of Dugda District, Central Rift Valley, Ethiopia. J. Ethnobiol. Ethnomed..

[10] Eiki N., Sebola N.A., Sakong B.M., Mabelebele M. (2021). Review on ethnoveterinary practices in sub-Saharan Africa. Vet Sci.

[11] Tchetan E., Olounlade A.P., Houehanou T.D., Azando E.V.B., Kaneho J.A., Houinato M.R.B., Hounzangbe-Adote S.M., Quetin-Leclercq J., Gbaguidi F.A. (2021). Ethnoveterinary knowledge of sheep and goat farmers in Benin (West Africa): Effect of socioeconomic and environmental factors. Heliyon.

[12] Onwubiko J.I., Igwillo U.C., Mbaoji C.O. (2020). Review of ethnoveterinary medicine for animal healthcare in Nigeria. Int. J. Rec. Res. Life Sci..

[13] Koné W.M., Atindehou K.K. (2008). Ethnobotanical inventory of medicinal plants used in traditional veterinary medicine in Northern Côte d'Ivoire (West Africa). South Afr. J. Bot..

[14] Barbosa F.M., Cala A.C., Sevastyanov V., Boane E., Hlashwayo D.F. (2023). Ethnoveterinary study of plant‐based remedies for treating diseases in small ruminants in Maputo province, Mozambique. Evid. Based Complement Alternat. Med.

[15] Mthi S., Rust J., Tokozwayo S., Dubeni Z.B. (2023). Ethnopharmacological assessment of medicinal plants used in the management of livestock ailments by resource-limited farmers in the Eastern Cape Province. Open J. Vet. Med..

[16] Eiki N., Maake M., Lebelo S., Sakong B., Sebola N., Mabelebele M. (2022). Survey of ethnoveterinary medicines used to treat livestock diseases in Omusati and Kunene regions of Namibia. Front. Vet. Sci..

[17] Traoré L., Yaro V.S.O., Soudre A., Ouedraogo-Kone S., Ouedraogo D., Yougbare B., Zoma B.L., Hien M., Guissou M.L., Traore A., Sölkner J. (2020). Indigenous knowledge of veterinary medicinal plant use in cattle treatment in southwestern Burkina Faso (West Africa). South Afr. J. Bot..

[18] Gao H., Huang W., Zhao C., Xiong Y. (2024). An ethnoveterinary study on medicinal plants used by the Bai people in Yunlong County northwest Yunnan, China. J. Ethnobiol. Ethnomed..

[19] Babacan E.Y., Polat R., Güler O., Moyan A., Paksoy M.Y., Cakilcioglu U. (2022). An ethno-veterinary study on plants used for the treatment of livestock diseases in Genç (Bingöl-Turkey). Indian J. Tradit. Knowl..

[20] Güler O., Polat R., Karaköse M., Çakılcıoğlu U., Akbulut S. (2021). An ethnoveterinary study on plants used for the treatment of livestock diseases in the province of Giresun (Turkey). South Afr. J. Bot..

[21] Mertenat D., Dal Cero M., Vogl C.R., Ivemeyer S., Meier B., Maeschli A., Hamburger M., Walkenhorst M. (2020). Ethnoveterinary knowledge of farmers in bilingual regions of Switzerland–is there potential to extend veterinary options to reduce antimicrobial use?. J. Ethnopharmacol..

[22] Awuni F. (2020). http://www.prolinnova.net/sites/default/files/documents/ghana/2019/ghana_ethnovet_project_report_oct_2019-march_2020_finalrev2.pdf.

[23] Alenyerege B., Mensah K. (2015). Incidence of retained placenta in ruminants and its treatment by rural farmers in northern Ghana. Indian J. Appl. Res..

[24] Octhere G.V., Naandam J. (2015). Effect of pounded Dawadawa (*Parkia biglobosa*) pod husk extract on strongyle in West African Dwarf (Wad) goats. UDS International Journal of Development.

[25] Nchor J. (2011).

[26] Turkson P.K., Naandam J. (2002). Traditional veterinary knowledge and practices in Northern Region of Ghana. Ghana J. Agric.

[27] Naandam J., Idorisu R. (2010). Effect of *Parkia biglobosa* (Dawadawa) pod extracts on strongyle ova in sheep. Anim. Res. Int.

[28] Morvin Yabesh J.E., Prabhu S., Vijayakumar S. (2014). An ethnobotanical study of medicinal plants used by traditional healers in silent valley of Kerala, India. J. Ethnopharmacol..

[29] Byg A., Balslev H. (2001). Diversity and use of palms in Zahamena, eastern Madagascar. Biodivers. Conserv..

[30] Albuquerque U.P., Lucena R.F.P., Monteiro J.M., Florentino A.T.N., Almeida C.F.C.B.R. (2006). Evaluating two quantitative ethnobotanical techniques. Ethnobot. Res. Appl..

[31] Ameleke G.Y., Haagsma R., Karbo N., Mensah-Bonsu A. (2020). The nature and drivers of contracts in cattle herding and management: the case of Ghana. Pastoralism.

[32] Ghana Statistical Service (2014). https://www2.statsghana.gov.gh/docfiles/2010_District_Report/Volta/ADAKLU.pdf.

[33] Ghana Statistical Service (2021). https://statsghana.gov.gh/gssmain/fileUpload/pressrelease/2021PHCGeneralReportVol3A_PopulationofRegionsandDistricts_181121.pdf.

[34] Boakye M.K., Agyemang A.O., Gbadegbe R.S., Quashie M., Turkson B.K., Adanu K.K., Wiafe E.D. (2023). Ethnobotanical applications of *Spathodea campanulata* P. Beauv. (African tulip tree) in Ghana. Ethnobot. Res. Appl..

[35] Boakye M., Adanu S.K., Akumah A.M., Buami E.K., Agyemang A.O. (2024). Plants used for ethnoveterinary treatment of free-range indigenous chicken diseases in Ghana. Ethnobot. Res. Appl..

[36] Abbiw D.K. (1990).

[37] Irvine F.R. (1961).

[38] Prance G.T., Baleé W., Boom B.M., Carneiro R.L. (1987). Quantitative ethnobotany and the case for conservation in Ammonia. Conserv. Biol..

[39] Tardío J., Pardo-de-Santayana M. (2008). Cultural importance indices: a comparative analysis based on the useful wild plants of Southern Cantabria (Northern Spain). Econ. Bot..

[40] Trotter R.T., Logan M.H., Etkin N.L. (1986). Plants in Indigenous Medicine and Diet.

[41] Ansah T., Nagbila D.A. (2011). Utilization of local trees and shrubs for sustainable livestock production in the Talensi-Nabdam District of the Upper East Region of Ghana. Livest. Res. Rural Dev..

[42] Lulekal E., Asfaw Z., Kelbessa E., Van Damme P. (2014). Ethnoveterinary plants of Ankober district, north Shewa zone, Amhara region, Ethiopia. J. Ethnobiol. Ethnomed..

[43] Adeniran L.A., Okpi S., Anjorin T.S., Ajagbonna O.P. (2020). Medicinal plants used in ethnoveterinary practices in the Federal Capital Territory, north-Central Nigeria. J. Med. Plants Res..

[44] Ayeni E.A., Basiri B. (2018). Ethnoveterinary survey of plants used in treating livestock among the Fulani people of Girei, Adamawa State, Nigeria. World News of Natural Sciences.

[45] Tekle Y. (2015). Medicinal plants in the ethno veterinary practices of Bensa woreda, Southern Ethiopia. OALibJ.

[46] Ouachinou J.A., Dassou G.H., Idohou R., Adomou A.C., Yédomonhan H. (2019). National inventory and usage of plant-based medicine to treat gastrointestinal disorders with cattle in Benin (West Africa). South Afr. J. Bot..

[47] Offiah N.V., Makama S., Elisha I.L., Makoshi M.S., Gotep J.G., Dawurung C.J., Oladipo O.O., Lohlum A.S., Shamaki D. (2011). Ethnobotanical survey of medicinal plants used in the treatment of animal diarrhoea in Plateau State, Nigeria. BMC Vet. Res..

[48] Reddy M.T., Kalpana M., Sivaraj N., Kamala V., Pandravada S.R., Sunil N. (2019). Indigenous traditional knowledge on health and equitable benefits of oil palm (*Elaeis* spp.). OALibJ.

[49] Owoyele B.V., Owolabi G.O. (2014). Traditional oil palm (*Elaeis guineensis* Jacq.) and its medicinal uses: a review. Tang [Humanitas Medicine].

[50] Kasali F.M., Tusiimire J., Kadima J.N., Tolo C.U., Weisheit A., Agaba A.G. (2021). Ethnotherapeutic Uses and Phytochemical Composition of *Physalis peruviana* L.: an Overview. Sci. World J..

[51] Rossato S.C., Leitão-Filho H.D.F., Begossi A. (1999). Ethnobotany of caiçaras of the Atlantic forest coast (Brazil). Econ. Bot..

[52] Boakye M.K., Wiafe E.D., Ziekah M.Y. (2021). Ethnomedicinal use of pythons by traditional medicine practitioners in Ghana. Afr. J. Herpetol..

[53] Heinrich M., Ankli A., Frei B., Weimann C., Sticher O. (1998). Medicinal plants in Mexico: healers' consensus and cultural importance. Soc. Sci. Med..

[54] Heinrich M., Edwards S., Moerman D.E., Leonti M. (2009). Ethnopharmacological field studies: a critical assessment of their conceptual basis and methods. J. Ethnopharmacol..

[55] Thomas E., Vandebroek I., Sanca S., Van Damme P. (2009). Cultural significance of medicinal plant families and species among Quechua farmers in Apillapampa, Bolivia. J. Ethnopharmacol..

[56] Constant N.L., Tshisikhawe M.P. (2018). Hierarchies of knowledge: ethnobotanical knowledge, practices and beliefs of the Vhavenda in South Africa for biodiversity conservation. J. Ethnobiol. Ethnomed..

[57] Reyes-García V., Broesch J., Calvet-Mir L., Fuentes-Peláez N., McDade T.W., Parsa S., Huanca T., Leonard W.R., Martínez-Rodríguez M.R., TAPS Bolivian Study Team (2009). Cultural transmission of ethnobotanical knowledge and skills: an empirical analysis from an Amerindian society. Evol. Hum. Behav..

[58] Appiah K.S., Oppong C.P., Mardani H.K., Omari R.A., Kpabitey S., Amoatey C.A., Onwona-Agyeman S., Oikawa Y., Katsura K., Fujii Y. (2018). Medicinal plants used in the Ejisu-Juaben municipality, southern Ghana: an ethnobotanical study. Medicines (Basel, Switzerland).

[59] Abel C., Busia K. (2005). An exploratory ethnobotanical study of the practice of herbal medicine by the Akan peoples of Ghana. Alternative Med. Rev..

[60] Yeboah T. (2000). Improving the provision of traditional health knowledge for rural communities in Ghana. Health Libr. Rev..

[61] Oyeyemi I.T., Akinlabi A.A., Adewumi A., Aleshinloye A.O., Oyeyemi O.T. (2018). *Vernonia amygdalina*: a folkloric herb with anthelminthic properties. Beni-Suef Univ. J. Basic Appl. Sci..

[62] Leonidas M., Faye D., Justin K.N., Viateur U., Angélique N. (2013). Evaluation of the effectiveness of two medicinal plants *Vernonia amygdalina* and *Leonotis nepetaefolia* on the gastrointestinal parasites of goats in Rwanda: case study of Huye and Gisagara districts. J. Vet. Med. Anim. Health.

[63] Yeap S.K., Ho W.Y., Beh B.K., Liang W.S., Ky H., Yousr A.H.N., Alitheen N.B. (2010). *Vernonia amygdalina*, an ethnoveterinary and ethnomedical used green vegetable with multiple bioactivities. J. Med. Plants Res..

